# Case Report and Literature Review of Prosthetic Cardiovascular Mucormycosis

**DOI:** 10.3201/eid2911.230837

**Published:** 2023-11

**Authors:** Baptiste Hoellinger, Louis Magnus, Yvon Ruch, Mickael Ohana, Yves Hansmann, Valérie Letscher-Bru, Anne Lejay, Nabil Chakfé, François Danion

**Affiliations:** University Hospital of Strasbourg, Strasbourg, France (B. Hoellinger, L. Magnus, Y. Ruch, M. Ohana, Y. Hansmann, V. Letscher-Bru, A. Lejay, N. Chakfé, F. Danion);; University Hospital of Clermont-Ferrand, Clermont-Ferrand, France (L. Magnus);; Inserm UMR_S 1109, Strasbourg (F. Danion)

**Keywords:** mucormycosis, fungi, fungal infection, Mucorales, prosthetic vascular graft infection, endocarditis, France, Candida albicans, Lactobacillus plantarum, Rhizopus microsporus, *Suggested citation for this article*: Hoellinger B, Magnus L, Ruch Y, Ohana M, Hansmann Y, Letscher-Bru V, et al. Case report and literature review of prosthetic cardiovascular mucormycosis. Emerg Infect Dis. 2023 Nov [*date cited*]. https://doi.org/10.3201/eid2911.230837

## Abstract

We report a rare case of aorto-bi-iliac prosthetic allograft mucormycosis in a 57-year-old immunocompetent patient in France. Outcome was favorable after surgery and dual antifungal therapy with liposomal amphotericin B and isavuconazole. In a literature review, we identified 12 other cases of prosthetic vascular or heart valve mucormycosis; mortality rate was 38%.

Mucormycosis, caused by fungi of order Mucorales, is a rare, life-threatening fungal infection whose incidence has been rising since the late 1990s (*1*). The main infection locations are pulmonary, rhino-orbito-cerebral, cutaneous, and disseminated. Although the vascular tropism of Mucorales is well described, few cases of cardiovascular infections have been reported (*2*). We report a rare case of aorto-bi-iliac prosthetic allograft mucormycosis in a 57-year-old immunocompetent patient in France. We obtained written informed consent from the patient for publication of this report.

The patient, who had a history of type B aortic dissection, underwent an open surgical repair of a right common iliac artery aneurysm with aorto-bi-iliac prosthetic graft reconstruction (day 0). We noted a bowel perforation at the end of the surgery and performed resection-anastomosis. Because of a history of allergy to penicillin, we treated the patient with aztreonam, metronidazole, vancomycin, and amikacin. The patient acquired an early postoperative *Candida albicans* infection diagnosed on periprosthetic collection puncture and treated with caspofungin on day 10. On day 30, he had emergency surgery for proximal anastomosis rupture with hemorrhagic shock (Figure, panels A, B). All the prosthetic material was excised with in situ reconstruction using a silver-coated prosthetic aorto-bi-iliac graft. Three intraoperative samples were positive for *Lactobacillus plantarum* and *Rhizopus microsporus* pathogens. Serum samples were positive by Mucorales PCR for *Rhizopus*, which we confirmed on 5 other samples (*3*). Histology was not performed. Neither chest computed tomography nor brain magnetic resonance imaging showed another location of infection. We replaced caspofungin with liposomal amphotericin B (5 mg/kg). 

**Figure:**
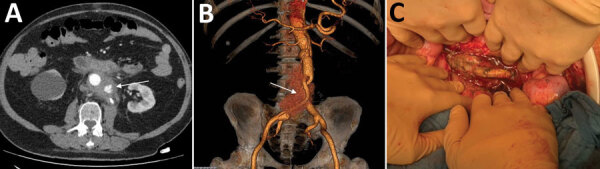
Vascular prosthetic mucormycosis in a 57-year-old immunocompetent patient in France. A, B) Aortic computed-tomography angiogram (A) and 3-dimensional reconstruction (B) show the periprosthetic collection and vascular leak (arrows). C) Intraoperative view shows the graft exposure and false necrotic membranes.

We performed surgical revision for recurrence of collection on day 37; the patient had retroperitoneal necrosis with false necrotic membranes, another digestive fistula, and graft exposure (Figure, panel C). We performed tissue debridement, perigraft collection drainage, and irrigation associated with bowel resection-anastomosis and omentoplasty to cover the graft. We administered isavuconazole with liposomal amphotericin B after surgery. Cultures of the peroperative samples found *R. microsporus* and *C. albicans*. Histology showed signs of acute inflammation in contact with the prosthetic fibers, but specific staining was not performed. Serum samples tested by Mucorales PCRs 3 times/week were still positive at day 37 and became negative at day 52. We performed surgery again, excising the silver-coated graft and then using a cryopreserved human allograft for in situ aorto-iliac reconstruction, on day 95. Three months later, the patient’s clinical and biologic progress was favorable; amphotericin B was discontinued, and isavuconazole was continued on a long-term basis. After 1 year of follow-up, the infection had not recurred. 

We performed a literature review of cases of prosthetic vascular or heart valve mucormycosis and identified 13 cases, including our case (Appendix). Nine of those patients were male and 4 female; median age was 54 years. Two (15%) of the patients had known immunosuppression, 1 from solid organ transplantation and 1 from hematologic malignancy. Two patients had received steroids in the weeks before illness. Seven patients had a vascular infection. Eight had endocarditis; of interest, 4 of those 8 patients had emboli in the lower limbs, which is usually a rare embolic site in endocarditis (*4*). Nine (69%) of 13 patients had an early postoperative infection (<4 months after surgery). The mucormycosis infection was monomicrobial in 10 (85%) of the 13 cases. Two patients were co-infected with *Aspergillus*; the patient we report was co-infected with *Candida albicans*. Ten of the 13 patients received treatment with liposomal or deoxycholate amphotericin B; 2 patients died before they could receive any treatment. Surgery was performed in 11/13 patients, and infected prothesis were explanted in 10 patients. Five (38%) of the 13 patients died.

The main fungal cause of vascular infection and endocarditis is *Candida* spp. Mucormycosis occurs mainly in immunocompromised patients. However, certain forms can occur in immunocompetent patients, particularly posttraumatic and healthcare-associated forms; prosthetic mucormycosis also seems to fall into this category (*5*). We suggest 2 hypotheses for the mechanism of mucormycosis in our patient: a healthcare-associated mucormycosis, if we consider that the implanted prosthesis could have been contaminated, or a contamination of the prosthesis by digestive perforation (*6*). As for the second hypothesis, Mucorales are found on many foods; in our patient’s case, ongoing treatment with caspofungin and broad-spectrum antimicrobial therapy could have encouraged colonization (*7*).

Our study and review of the literature suggest a better prognosis for vascular prosthetic mucormycosis than for pulmonary and disseminated mucormycosis, probably because it occurs in immunocompetent patients and the source can be effectively controlled by surgery. However, we acknowledge that reporting cases with favorable outcomes may have introduced bias. The first-line treatment for mucormycosis is liposomal amphotericin B (5 mg/kg) (*8*). Surgery is crucial for controlling the infection, particularly in extrapulmonary locations (*8*). The benefit of amphotericin B/isavuconazole dual therapy has been suggested in a neutropenic mice model and should be explored for difficult-to-treat locations, especially when a prosthesis is involved (*9*). In conclusion, prosthetic cardiac and vascular mucormycosis are very rare infections that require prompt surgery and antifungal therapy.

Appendix Literature review for cases of prosthetic cardiovascular mucormycosis.
