# Supplementary material for: Case Report and Literature Review of Prosthetic Cardiovascular Mucormycosis
Source: Emerg Infect Dis. 2023 Nov;29(11):2388–90. doi: 10.3201/eid2911.230837 (PMC10617327; doi:10.3201/eid2911.230837)
Supplement: Appendix — Literature review for cases of prosthetic cardiovascular mucormycosis. [file 23-0837-Techapp-s1.pdf]

Article DOI: http://doi.org/10.3201/eid2911.230837

EID cannot ensure accessibility for supplementary materials supplied by authors. Readers who have difficulty accessing supplementary content should contact the authors for assistance.

Case Report and Literature Review of Prosthetic Cardiovascular Mucormycosis

Appendix

Appendix Table. Characteristics of patients with cardiovascular mucormycosis from current case and literature review*

Patient and ref	Sex, Age	Background	Site of infection.	Delay†	Mucorales species	Co-infection	Antifungal treatment	Surgery	Outcome
P1<br>This case	M, 57	Type B aortic dissection	Aorto-bi-iliac bypass	Mo1	*Rhizopus microspores (DE, culture)*	*Candida albicans, Lactobacillus plantarum*	L-AmB + IZV<br>Lifelong IZV	First surgery: Bypass removal for Silver prosthesis.<br>D15 : Resection of a digestive fistula + lavage + omentoplasty<br>W6: Aorto bifemoral arterial allograft	Survived (M12)<br>No recurrence
P2 (*1*)	M, 57	Type A aortic dissection	Ascending aortic and brachiocephalic prosthetic graft	NA	*Rhizopus arrhizus (Histology)*	No	L-AmB	Removal of a mass<br>Aortic and brachiocephalic artery graft replacement	Survived (M9)<br>No recurrence
P3 (*2*)	M, 38	Pseudoaneurysm of right iliac artery: stent<br>Renal transplantation cyclosporine and steroids<br>Acute reject due to arterial interruption<br>Nephrectomy	Iliac artery stent	Mo2	Mucorales *(Histology)*	No	L-AmB<br>3 mo	Stent removal. Suprapubic femoral-femoral bypass with homologous saphenous vein graft	Survived (M12)<br>No recurrence

Patient and ref	Sex, Age	Background	Site of infection.	Delay†	Mucorales species	Co-infection	Antifungal treatment	Surgery	Outcome
P4 (*3*)	F, 22	Coarctation of aorta	Dacron aortic graft	D18	*Mucor sp. (Histology, DE, culture)*	No	d-AmB 7 weeks	Dacron graft replacement	Survived (M12) No recurrence.
P5 (*4*)	F, 60	Aortobifemoral bypass	Saphenous vein graft	D9	Mucorales *(Histology, DE, negative culture)*	No	d-AmB	Replacement by new saphenous vein graft	Death
P6 (*5*)	M, 55	Ascending aortic aneurism Ocular histoplasmosis	Native aortic valve + dacron ascending aortic graft + splenic abscess	Y4	*Rhizopus sp. (DE, culture)*	*Aspergillus sp.*	PCZ (intolerance to AmB)	Cadaveric aortic homograft	Survived (M12) No recurrence
P7 (*6*)	M, 60	Degenerative aortic valve disease	Mechanical aortic valve + Septic emboli left lower limb	Mo1.5	*Mucor sp. (Histology, DE, culture)*	No	d-AmB + ICZ, 6–8 weeks	Prosthetic valve replacement	Survived (M12) No récurrence
P8 (*7*)	F, 80	3rd degree AV Block Essential thrombocytosis treated with hydroxyurea, COPD, ischemic strock	Ventricular pacemaker lead + native tricuspid valve + septic embolic right lower limb	Y3	*Mucor sp. (DE, culture)*	No	L-AmB 7mg/kg	Removal of the entire pacemaker system	Death
P9 (*8*)	M, 84	Coronary artery bypass grafting, aortic valve replacement, pacemaker for post-operative heart block	Bioprosthetic aortic valve	Mo2	*Lichtheimia sp. (DE, culture)*	No	L-AmB + FCZ then lifelong PCZ	First aortic valve replacement surgery, no change of PM leads Second surgery at M7 for recurrence	Survived. Recurrence with negative perioperative samples (M7)
P10 (*9*)	M, 52	Mitral stenosis. Steroids	Mitral mechanical valve	D25	*Mucor sp.* (DE, culture)	No	Early death	Died before	Death
P11 (*10*)	F, 18	Mitral regurgitation Tricuspid annuloplasty for tricuspid regurgitation	Mechanical mitral valve + Bilateral femoral emboli	Mo3	*Mucor sp. (Histology, DE, culture)*	No	AmB	Vegetation removal + local instillation of amphotericin B	Survived. No recurrence
P12 (*11*)	M, 47	Aortic valvulopathy	Mechanical aortic valve + femoral artery emboli	Mo3	Mucor sp. *(Blood cultures, histology, culture)*	*Aspergillus sp.*	d-AmB	Died before	Death

Patient and ref	Sex, Age	Background	Site of infection.	Delay†	Mucorales species	Co-infection	Antifungal treatment	Surgery	Outcome
P13 (*12*)	M, 50	Aortic Stenosis. Steroids for pericarditis	Mechanical aortic valve. Ascending aorta (Saccular aneurysm)	Mo7	Mucorales (Histology)	No	Died before	Peri-operative rupture of the aneurysm	Death

*AmB, amphotericin B; d-AmB, deoxycholate amphotericin B; L-AmB, liposomal amphotericin B; D, day; DE, direct examination; F, female; FCZ, fluconazole; ICZ, itraconazole; IZV, isavuconazole; M, male; Mo, month; NA, non available; Y, year; PCZ, posaconazole.
†Delay between the implantation of the material and the symptoms.

References

1. Zhou X, Song L, Jin J. Ascending aortic prosthetic graft mucormycosis. Eur Heart J. 2022;43:4213. PubMed https://doi.org/10.1093/eurheartj/ehac422

2. Liapis CD, Petrikkos GL, Paraskevas KI, Skiada A, Nikolaou AC, Tzortzis G, et al. External iliac artery stent mucormycosis in a renal transplant patient. Ann Vasc Surg. 2006;20:253–7. PubMed https://doi.org/10.1007/s10016-006-9007-1

3. Callard GM, Wright CB, Wray RC, Minor GR. False aneurysm due to Mucor following repair of a coarctation with a dacron prosthesis. J Thorac Cardiovasc Surg. 1971;61:181–5. https://doi.org/10.1016/S0022-5223(19)42245-9

4. Thomford NR, Dee TH, Sherman NJ, Klainer AS. Mucormycosis of a saphenous vein autograft. Arch Surg. 1970;101:518–9. PubMed https://doi.org/10.1001/archsurg.1970.01340280070018

5. Nebel C, Gersch K, Smith JM, Rodersheimer LR, Friedstrom S. Successful treatment of ascending aortic graft and valve endocarditis after debulking splenectomy for invasive aspergillosis and mucor. Am Surg. 2009;75:1253–5. PubMed https://doi.org/10.1177/000313480907501221

6. Sanchez-Recalde A, Merino JL, Dominguez F, Mate I, Larrea JL, Sobrino JA. Successful treatment of prosthetic aortic valve mucormycosis. Chest. 1999;116:1818–20. PubMed https://doi.org/10.1378/chest.116.6.1818

7. Metallidis S, Chrysanthidis T, Kazakos E, Saraf A, Nikolaidis P. A fatal case of pacemaker lead endocarditis caused by *Mucor* spp. Int J Infect Dis. 2008;12:e151–2. PubMed https://doi.org/10.1016/j.ijid.2008.03.034

8. Terrien CM III, Edwards NM. Aortoventricular dissociation and refractory fungal endocarditis caused by a rare pathogen *Lichtheimia*: a surgical and medical management strategy. Ann Thorac Surg. 2017;103:e25–7. PubMed https://doi.org/10.1016/j.athoracsur.2016.06.036

9. Khicha GJ, Berroya RB, Escano FB Jr, Lee CS. Mucormycosis in a mitral prosthesis. J Thorac Cardiovasc Surg. 1972;63:903–5. PubMed https://doi.org/10.1016/S0022-5223(19)41816-3

10. Chaudhry R, Venugopal P, Chopra P. Prosthetic mitral valve mucormycosis caused by Mucor species. Int J Cardiol. 1987;17:333–5. PubMed https://doi.org/10.1016/0167-5273(87)90085-4

11. Mishra B, Mandal A, Kumar N. Mycotic prosthetic-valve endocarditis. J Hosp Infect. 1992;20:122–5. PubMed https://doi.org/10.1016/0195-6701(92)90115-3

12. Rainer WG, Liggett MS, Quianzon EP, Dirks DW. Infected aortotomy due to Mucor following aortic valve replacement. J Thorac Cardiovasc Surg. 1970;59:781–4. PubMed https://doi.org/10.1016/S0022-5223(19)42398-2
